# Classification of Gait Patterns in Patients with Neurodegenerative Disease Using Adaptive Neuro-Fuzzy Inference System

**DOI:** 10.1155/2018/9831252

**Published:** 2018-09-30

**Authors:** Qiang Ye, Yi Xia, Zhiming Yao

**Affiliations:** ^1^Department of Sport and Health Science, Nanjing Sport Institute, Nanjing 210014, China; ^2^School of Electrical Engineering and Automation, Anhui University, Hefei 230601, China; ^3^Institute of Intelligent Machines, Chinese Academy of Sciences, Hefei 230031, China

## Abstract

A common feature that is typical of the patients with neurodegenerative (ND) disease is the impairment of motor function, which can interrupt the pathway from cerebrum to the muscle and thus cause movement disorders. For patients with amyotrophic lateral sclerosis disease (ALS), the impairment is caused by the loss of motor neurons. While for patients with Parkinson's disease (PD) and Huntington's disease (HD), it is related to the basal ganglia dysfunction. Previously studies have demonstrated the usage of gait analysis in characterizing the ND patients for the purpose of disease management. However, most studies focus on extracting characteristic features that can differentiate ND gait from normal gait. Few studies have demonstrated the feasibility of modelling the nonlinear gait dynamics in characterizing the ND gait. Therefore, in this study, a novel approach based on an adaptive neuro-fuzzy inference system (ANFIS) is presented for identification of the gait of patients with ND disease. The proposed ANFIS model combines neural network adaptive capabilities and the fuzzy logic qualitative approach. Gait dynamics such as stride intervals, stance intervals, and double support intervals were used as the input variables to the model. The particle swarm optimization (PSO) algorithm was utilized to learn the parameters of the ANFIS model. The performance of the system was evaluated in terms of sensitivity, specificity, and accuracy using the leave-one-out cross-validation method. The competitive classification results on a dataset of 13 ALS patients, 15 PD patients, 20 HD patients, and 16 healthy control subjects indicated the effectiveness of our approach in representing the gait characteristics of ND patients.

## 1. Introduction

Neurodegeneration is the progressive loss of structure or function of neurons, including death of neurons. Many neurodegenerative diseases, such as amyotrophic lateral sclerosis (ALS), Parkinson's disease (PD), and Huntington's disease (HD), occur as a result of neurodegenerative processes. Such diseases are incurable, resulting in progressive degeneration and/or death of neuron cells and producing changes in altered neuromuscular control. Since flexion and extension motions of two lower limbs are regulated by the central nervous system, the gait of a patient with a neurodegenerative disorder would become abnormal due to deterioration of motor neurons [1]. PD and HD are two neurodegenerative disorders of the basal ganglia, and the ability to maintain a steady walk with small stride-to-stride fluctuations is dramatically impaired [2]. Amyotrophic lateral sclerosis (ALS), which is also referred to as Charcot's disease, is a progressive neuromuscular disease caused by the destruction of motor neurons in the brain and spinal cord [3]. This causes loss of nervous control of the voluntary muscles, resulting in the degeneration and atrophy of the muscles. At the early stage of ALS, weakness of lower motor neurons would affect the movement function and may lead to balanced impairment or altered gait rhythm [2, 4]. Therefore, analysis of gait cadence may help us to produce automatic noninvasive detection of movement disorders caused by the degeneration of motor neurons.

Several studies have analysed the impact of ND diseases on the gait dynamics of the subjects. Hausdorff et al. [5] found that the ability to maintain a steady gait, with low stride-to-stride variability of gait cycle timing and its subphases, was diminished for both PD and HD, two typical disorders of basal ganglia. Furthermore, they found that gait speed was significantly lower in PD, but not in HD, and average gait cycle duration and the time spent in many subphases of the gait cycle were similar in control (CO) subjects, HD subjects, and PD subjects. In another similar study, Hausdorff et al. [2] studied the magnitude of the stride-to-stride fluctuations and perturbations of the gait rhythm in subjects with ALS disease in comparison with healthy CO subjects and PD and HD patients. Their study revealed a longer stride interval for ALS patients than all the other three compared groups. They also found that patients with ALS have less steady gait between successive stride intervals in comparison with control subjects, using the detrended fluctuation analysis technique.

In order to facilitate automated identification of neurodegenerative diseases like PD, HD, and ALS for the clinical assistant purpose by using gait signals, different machine learning tools have also been applied in previous studies. Baratin et al. [6] introduced a wavelet-based scheme for effective characterization of gait associated with ALS patients. They extracted features such as entropy that reflects the regularity of gait and coherence between left and right limbs from the wavelet approximation part of the raw gait signal. Then, the classification of ALS gaits and normal gaits was implemented with a linear discriminant analysis (LDA) classifier. Wu et al. [7] quantified the gait variability in patients with PD by counting the signal turns. They found that the signal turns in the stride interval time series presented a significant difference between the healthy control subjects and the PD patients. In another similar study by the same authors [8], the nonparametric Parzen window method was utilized to estimate the probability density functions (PDFs) of gait stride interval time series, and then by computing the mean of left foot stride interval time series and the modified Kullback–Leibler divergence of the PDFs between left foot and right foot as the two features, they realized the classification of gait in subjects with ALS and normal subjects by using the least squares support vector machines (LS-SVM).

These studies suggest that gait cadence exhibits complex and nonlinear behaviour in both patients with ND diseases and control (CO) subjects because of the nonlinear dynamics of the human system [9, 10]. However, due to the presence of neurological disorders, the ND patients' gait rhythm manifests significant differences than that of the healthy CO subjects, such as the magnitude of stride-to-stride gait variability, gait speed [2], and the degree of asymmetry in gait rhythm [11]. Previous evidence has shown that discrimination between normal and abnormal gait could be implemented by means of the statistical, nonlinear computational method [1, 6, 8, 12]. ANFIS harnesses the power of two paradigms, fuzzy logic and artificial neural networks, which have shown significant results in modelling nonlinear functions [13, 14]. In the ANFIS, the membership function parameters are learned from a dataset that describes the system behaviour under the constraints of a given error criterion [15, 16]. As far as we know, ANFIS has been successfully applied in biomedical engineering for different tasks such as classification [17, 18] and data analysis [19].

In this study, a novel approach based on ANFIS is presented for the classification of gait cadence between the patients with ND disease and the healthy subjects. The ANFIS model combined with the particle swarm optimization (PSO) algorithm is used to learn the nonlinear gait dynamics by using five gait cadence signals as the input variables, and then the classification between patients with ALS disease and normal subjects is implemented with a simple distance-based classifier by using the outputs of the ANFIS model. This paper has been organized as follows: the description of the gait dataset and an overview of our proposed method are given in Section 2. The experimental results and some discussions are presented in Section 3. Finally, some conclusions are given in Section 4.

## 2. Methods

### 2.1. Description of Dataset

The gait dataset used in this study was contributed by Hausdorff et al. [2], and it has been used by several different studies [1, 6, 8] to investigate the gait characteristics of the patients with neurodegenerative diseases. To measure the gait signals, force-sensitive switches were placed in the subject's shoe. The gait signals were sampled at 300 Hz via an onboard analog-to-digital converter (12 bit). The experimental protocol required that each subject should walk at their normal pace along a 77 m-long hallway for 5 min. According to the descriptions in [2], the first 20 s of the recorded data were excluded to eliminate the start-up effects and a median filter was applied to remove data points that had a standard deviation (SD) greater than 3 or were less than the median value. As we had introduced in our previous study [20], the gait dataset used in this study provides the following seven gait parameters: left and right stride interval (time from initial contact of one foot to the immediate subsequent contact), left and right stance interval (amount of time when one foot is on the ground), left and right swing interval (amount of time when one foot is in the air), and double support interval (time of bilateral foot contact).

Thirteen ALS patients, fifteen PD patients, and twenty HD patients were recruited from the Neurology Outpatient Clinic at Massachusetts General Hospital, Boston, MA, USA. Also, another sixteen healthy CO subjects were enrolled to conduct the comparative study. Each subject provided informed consent as approved by the Institutional Review Board of the Massachusetts General Hospital. There was no significant difference between the weights and heights of the CO subjects and the ND patients. The ND patients were not using a wheelchair or assistive device for mobility and were free of other ailments that might affect lower extremity weakness. The presence or absence of symptoms that might affect the gait was determined by a qualified physician. The severity of ALS is represented by the time since the onset of the disease. The Hoehn and Yahr (H&Y) scale [21] and the total functional capacity (TFC) [22] are used to assess the degree of neurologic impairment in PD and HD patients, respectively. Basic information of all the subjects participated in this study has been listed in Table 1.

### 2.2. Adaptive Neuro-Fuzzy Inference System (ANFIS)

#### 2.1.1. Architecture of ANFIS

The ANFIS is a useful neural network method for the solution of function approximation problems [23]. An ANFIS produces the mapping relation between the input and output data by using the hybrid learning method to determine the optimal distribution of membership functions. It combines artificial neural network (ANN) and fuzzy logic. Such a framework makes ANFIS modelling more systematic and less reliant on expert knowledge. To present the ANFIS architecture, for simplicity, two fuzzy if-then rules based on a first-order Sugeno model [15] can be stated as

(1) $$\begin{array}{c} \text{rule}1:\text{if }x\text{ is }A_{1}\text{ and }y\text{ is }B_{1}\text{ then }z\text{ is }f_{1}\left(x,y\right)=p_{1}x+q_{1}y+r_{1},\\ \text{rule}2:\text{if }x\text{ is }A_{2}\text{ and }y\text{ is }B_{2}\text{ then }z\text{ is }f_{2}\left(x,y\right)=p_{2}x+q_{2}y+r_{2}, \end{array}$$

where *x* and *y* are the inputs of ANFIS; *A*_*i*_ and *B*_*i*_ are the fuzzy sets; *f*_*i*_(*x*, *y*) is a first-order polynomial and represents the outputs of the first-order Sugeno fuzzy inference system; and *p*_*i*_, *q*_*i*_, and *r*_*i*_ are the design parameters that are determined during the training process.

**Figure 1:**
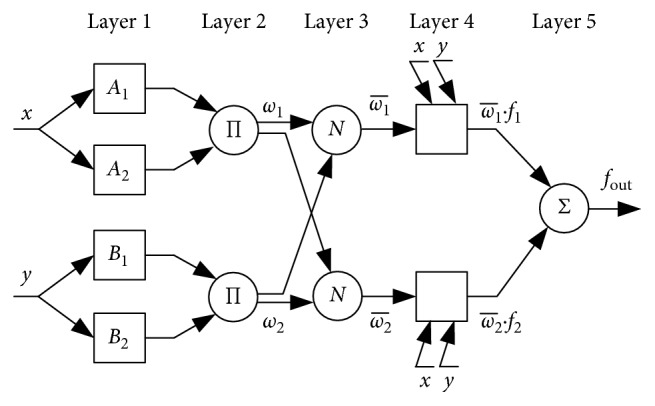
Architecture of the ANFIS with five layers.

The architecture of ANFIS is shown in Figure 1, and the node function in each layer is described below. Adaptive nodes, denoted by squares, represent the parameter sets that are adjustable in these nodes, whereas fixed nodes, denoted by circles, represent the parameter sets that are fixed in the system [23].

In the first layer, all the nodes are adaptive nodes. The output can be specified as

(2) $$\begin{array}{c} O_{1}^{i}=\mu_{{\text{A}}_{i}}\left(x\right),\;i=1,2,\\ O_{1}^{i}=\mu_{{\text{B}}_{i-2}}\left(y\right),\;i=3,4, \end{array}$$

where *μ*_A_*i*__(*x*) and *μ*_B_*i*−2__(*y*) can adopt any fuzzy membership function. For instance, if the bell-shaped membership function is applied, *μ*_A_*i*__(*x*) is given by

(3) $$\begin{array}{c} \mu_{{\text{A}}_{i}}\left(x\right)=\frac{1}{1+{\left\{{\left[\left(x-c_{i}\right)/a_{i}\right]}^{2}\right\}}^{b_{i}}}, \end{array}$$

where *a*_*i*_, *b*_*i*_, and *c*_*i*_ are the parameters set governing the bell-shaped functions accordingly. These parameters are named as premise parameters [23].

In the second layer, the nodes are fixed nodes marked by a circle. They are labeled Π, indicating that they serve as multipliers. The output of this layer can be given as

(4) $$\begin{array}{c} O_{2}^{i}=\omega_{i}=\mu_{{\text{A}}_{i}}\left(x\right)\cdot \mu_{{\text{B}}_{i}}\left(y\right)\;\text{for }i=1,2, \end{array}$$

where the output *ω*_*i*_ represents the firing strength of a rule.

Every node in the third layer is also a fixed node marked by a circle and labeled *N*, with the node function of normalizing the firing strengths of the previous layer. The outputs of this layer can be represented as

(5) $$\begin{array}{c} O_{3}^{i}={\bar{\omega }}_{i}=\frac{\omega_{i}}{\sum \omega_{i}}=\frac{\omega_{i}}{\omega_{1}+\omega_{2}}\;\text{for }i=1,2. \end{array}$$

In the fourth layer, the nodes are adaptive nodes. The output is simply calculated as

(6) $$\begin{array}{c} O_{4}^{i}={\bar{\omega }}_{i}\cdot f_{i}\;\text{for }i=1,2, \end{array}$$

where *f*_1_ and *f*_2_ are the fuzzy if-then rules mentioned before and parameters *p*_*i*_, *q*_*i*_, and *r*_*i*_ are also referred to as the consequent parameters.

The single node in fifth layer is a fixed node. This node performs the summation of all the incoming signals. The overall output of ANFIS is given as

(7) $$\begin{array}{c} O_{5}^{i}=f_{\text{out}}=\underset{i}{\sum }{\bar{\omega }}_{i}\cdot f_{i}=\frac{\omega_{1}f_{1}+\omega_{2}f_{2}}{\omega_{1}+\omega_{2}}, \end{array}$$

#### 2.1.2. Learning Algorithm of ANFIS

The aim of the learning algorithm for this architecture is to tune all the modifiable parameters, including {*a*_*i*_, *b*_*i*_, *c*_*i*_} and {*p*_*i*_, *q*_*i*_, *r*_*i*_}, so as to match the ANFIS output with the training data. In this study, ANFIS employs particle swarm optimization (PSO) algorithm to adjust the parameters of the membership functions [24]. The PSO techniques have the advantage of being less computationally expensive for a given size of network topology [25].

PSO is a swarm intelligence technique first introduced by Eberhart and Kennedy [26] that imitates the social behaviour of groups of insects and animals such as swarms of bees, flocks of birds, and shoals of fish [27]. Empirical evidence has been accumulated to show that the PSO algorithm is a useful tool for optimization, and it has been applied to many optimization problems in engineering [28, 29].

Suppose that the searching space is *D*-dimensional. A swarm of *N* particles are initialized in which each particle is assigned a random position in the *D*-dimensional hyperspace. Let *x* denotes a particle's position and *v* denotes the particle's flight velocity over a solution space.

The best historical position of a particle is Pbest. The global best historical position among all particles in the swarm is Gbest. Velocity and position of a particle are updated by the following rules:

(8) $$\begin{array}{c} \nu_{i}\left(t\right)=\omega \nu_{i}\left(t-1\right)+c_{1}\times {\mathrm{rand}}_{1}\left(\cdot \right)\times \left({x_{\text{Pbest}}}_{i}-x_{i}\left(t\right)\right)+c_{2}\times {\mathrm{rand}}_{2}\left(\cdot \right)\times \left(x_{\text{Gbest}}-x_{i}\left(t\right)\right),\\ x_{i}\left(t\right)=x_{i}\left(t-1\right)+\nu_{i}\left(t\right), \end{array}$$

where *ω* is the inertia weight; rand_1_(·) and rand_2_(·) are the uniformly distributed random numbers between 0 and 1; and *c*_1_ and *c*_2_ are the learning rates. In this work, constants *c*_1_ and *c*_2_ are both set at 2.0, which was suggested in [24]. Also, as suggested in the same work [24], an inertia correction function called “inertia weight approach (IWA)” is applied. During the IWA, the inertia weight *ω* is modified according to the following equation:

(9) $$\begin{array}{c} \omega =\omega_{\max}-\frac{\omega_{\max}-\omega_{\min}}{{\text{Itr}}_{\max}}\text{Itr}, \end{array}$$

where *ω*_max_ and *ω*_min_ are the initial and final inertia weights, Itr_max_ is the maximum number of iteration, and Itr is the current number of iteration.

The particle's fitness is calculated by inputting its position into a designed objective function. In this study, the optimization function to be minimized is root mean square error (RMSE), defined by the following equation:

(10) $$\begin{array}{c} \text{RMSE}=\sqrt{\frac{\sum_{k=1}^{N}{\left[z\left(k\right)-z_{\text{M}}\left(k\right)\right]}^{2}}{N}}, \end{array}$$

where *z*(*k*) is the *k*th actual sample value, *z*_M_(*k*) is the target output of the ANFIS model for the input features of the *k*th sample, and *N* is the amount of training samples.

### 2.3. Performance Evaluation

In the classification stage, for a test subject, a segment of gait dynamics is inputted to the ANFIS model, which will produce the same length of outputs. The average value of these outputs will be used to predict the final label of the test subject, which is determined according to the minimum distance of the average value to the label of either patients or healthy CO subjects. In our experiments, first each group of ND patients against the healthy CO subjects was evaluated, and then we evaluated all groups of neurodegenerative diseases against the healthy CO subjects. To validate the performance of the proposed method, the leave-one-out cross-validation (LOOCV) method over the entire dataset was done. During LOOCV, one subject was left out at a time and used for testing, and other remaining subjects were used as training data. The test performance of the ANFIS model was evaluated by the following statistical measures: specificity (*S*_p_), sensitivity (*S*_n_), and classification accuracy (*C*_a_).

(11) $$\begin{array}{c} S_{\text{n}}=\frac{\text{TP}}{\text{TP}+\text{FN}},\\ S_{\text{p}}=\frac{\text{TN}}{\text{TN}+\text{FP}},\\ C_{\text{a}}=\frac{\text{TN}+\text{TP}}{\text{TN}+\text{TP}+\text{FN}+\text{FP}}, \end{array}$$

where TP, TN, FP, and FN are true positives, true negatives, false positives, and false negatives, respectively.

## 3. Results and Discussion

In our experiments, the following five different gait parameters are chosen as the inputs to the ANFIS system: left and right stride interval, left and right stance interval, and double support interval. Also, as mentioned before, there are a few of walking turns that happened when the subjects reached the end of the hallway. In this study, only the longest time series segment between two adjacent walking turns is utilized. And every gait record in such segment is treated as an independent observation to be used as an input for training the ANFIS system. The statistics of each feature for different category of subjects is presented in Table 2.

**Figure 2:**
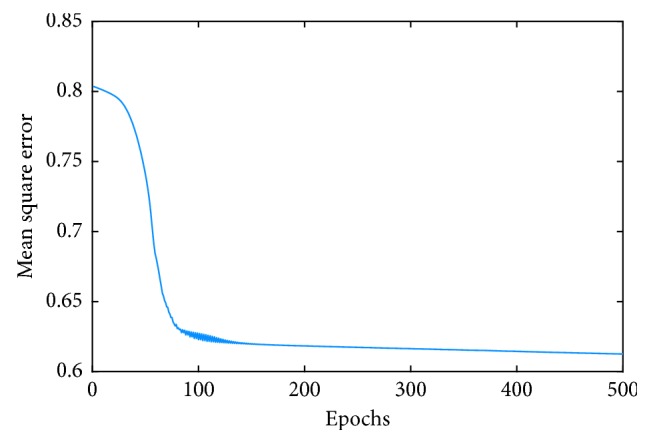
The curve of network error convergence of the ANFIS.

**Figure 3:**
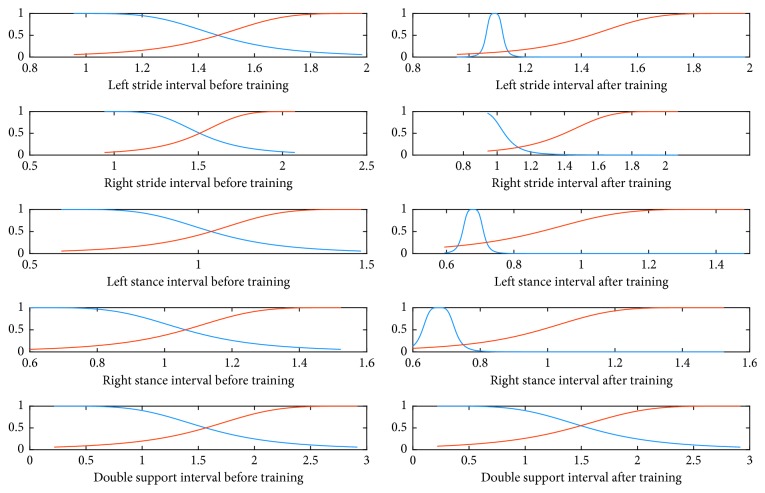
Generalized bell-shaped membership before and after training.

The final target outputs of ANFIS are designated as 0 and 4, indicating healthy CO subjects and ND patients, respectively. With five input variables and two membership functions each, the number of rules reaches 2^5^=32. As an example, the mean square error curve of training the ANFIS model for classification between the ALS patients and the CO subjects is shown in Figure 2. The initial and final membership functions are shown in Figure 3. Based on the analysis of membership functions of each input feature, it was found that most of them have considerable impact on the final identification of ALS patient.

The classification results between the ALS patients and the CO subjects are shown in Table 3. It can be observed that only one ALS patient and one CO subject were wrongly classified. Thus, the statistical measures of the classification performance evaluated by the LOOCV method are as follows: a specificity of 93.75%, a sensitivity of 92.31%, and an accuracy of 93.10%. The classification performances of PD patients vs. CO subjects and HD patients vs. CO subjects are listed in Tables 4 and 5, respectively. The accuracy for differentiating PD patients from CO subjects is 90.32%, with two PD patients and one CO subjects are wrongly classified. Similarly, in the experiments of diagnosing HD patients, 19 out of 20 HD patients and 15 out of 16 CO subjects are correctly recognized, leading to an accuracy of 94.44%. Finally, the results of all groups of neurodegenerative patients vs. CO subjects are reported in Table 6, where a specificity of 87.50%, a sensitivity of 91.67% and an accuracy of 90.63% are reported.

Furthermore, another experiment is performed to explore the performance of the proposed method in discriminating ND patients with different severity levels. According to the dividing approach proposed in [2], the ND gait data available in this study are broadly categorized into two groups: mild lower extremity functional impairment and more advanced functional impairment. The severe group consists of 9 PD patients with the Hoehn and Yahr score greater than or equal to 3, 9 HD patients with their total functional capacity score less than or equal to 5, and 9 ALS patients with stride time greater than 1.2 s. The rest belongs to the group with mild impairment. Discrimination accuracies were calculated for classification between the CO group and the two ND groups. The classification results are shown in Table 7. The best performance (an accuracy of 100%) was obtained for the classification between the CO group and the severe ND group. The accuracy for classifying the CO subjects and the mild ND group is 86.49%. However, for the classification between the two ND groups, i.e., mild ND group vs. severe ND group, the statistical measures of the performance of a sensitivity of 85.19%, a specificity of 80.95%, and an accuracy of 83.33% were still very promising. Such a result indicated that the proposed classification model could be used as a potential technique for identifying ND patients with different levels of severity.

The comparison of the classification performance between the proposed algorithm in this study and several state-of-the-art classification methods [1, 6, 7, 12, 30] on the same dataset is tabulated in Table 8. It can be noted that our proposed ANFIS-based method obtained the most accurate classification results in most cases. For example, for the purpose of identifying the ALS patients' gaits from the normal gaits, Wu et al. [1] extracted features like swing interval turns count and averaged stride interval. By using the least squares support vector machine (LS-SVM) as the classifier, they obtained an overall accuracy of 89.66%, which is a bit lower than an accuracy of 93.10% obtained in the present study. Zeng and Wang [12] modelled the gait dynamics with the radial basis function (RBF) neural networks, and by using the model learned via deterministic learning, they reported a classification accuracy of 87.1% between PD patients and CO subjects, while our method presented an accuracy of 90.32%. These competitive results indicate that the proposed approach can be effective for the classification of ND patients using gait dynamics.

However, as a limitation of this study, current gait dataset available at Physionet is small at its size. Therefore, for actual clinical purposes, such as gait monitoring of disease progression and evaluation of the response of therapy, more ND patients at different severities and age levels should be recruited into the database in the future studies. When more ND patients and matched CO subjects were enrolled into the study, the training of the ANFIS model could be more effective, and thus the proposed system would be more instructive for identifying the innate gait differences between ND patients and CO subjects.

## 4. Conclusions

In this study, a new classification scheme has been proposed for the purpose of classifying the gait of ND patients from normal gait. The presented ANFIS model combines neural network adaptive capabilities and the fuzzy logic qualitative approach, which provides an ideal tool for modelling the nonstationary human gait dynamics. Five gait cadence time series: left stride interval, right stride interval, left stance interval, right stance interval, and double support interval were used as the input variables of the ANFIS model. When evaluated with the LOOCV method, the classification accuracies of the ANFIS model for discriminating ND vs. CO, ALS vs. CO, PD vs. CO, and HD vs. CO groups were 90.63%, 93.10%, 90.22%, and 94.44%, respectively. By taking into consideration the misclassification rates, the proposed ANFIS model showed the potentials that help in identifying patients with ND from CO subjects by analyzing the gait data.

## Figures and Tables

**Table 1:** Some information about the subjects participated in the experiments.

Statistical parameter	Mean ± SD			
	CO	ALS	HD	PD
Age (year)	39.3 ± 18.5	55.6 ± 12.8	47.4 ± 12.5	66.8 ± 10.9
Height (m)	1.83 ± 0.08	1.74 ± 0.10	1.84 ± 0.09	1.87 ± 0.15
Weight (kg)	66.81 ± 11.08	77.11 ± 21.15	73.47 ± 16.23	75.07 ± 16.9
Gait speed (m/s)	1.35 ± 0.16	1.05 ± 0.22	1.15 ± 0.35	1.0 ± 0.2
Disease severity	0	18.3 ± 17.8	6.79 ± 3.9	2.8 ± 0.86

**Table 2:** The statistics of each feature for different groups of subjects.

Feature description	Mean ± SD			
	ALS	PD	HD	CO
Left stride interval (s)	1.520 ± 0.093	1.173 ± 0.052	1.146 ± 0.093	1.112 ± 0.083
Right stride interval (s)	1.520 ± 0.095	1.173 ± 0.046	1.146 ± 0.093	1.112 ± 0.077
Left stance interval (s)	1.043 ± 0.092	0.788 ± 0.047	0.763 ± 0.072	0.737 ± 0.073
Right stance interval (s)	1.023 ± 0.078	0.814 ± 0.052	0.800 ± 0.066	0.720 ± 0.060
Double support interval (s)	0.546 ± 0.080	0.429 ± 0.066	0.416 ± 0.071	0.345 ± 0.057

**Table 3:** Confusion matrix for the classification performance of the proposed system on differentiating ALS patients from the CO subjects.

Positive class (ALS)		Negative class (CO)		Specificity (*S*_p_)	Sensitivity (*S*_n_)	Accuracy (*C*_a_)
TP	FN	TN	FP			
12	1	15	1	93.75%	92.31%	93.10%

**Table 4:** Confusion matrix for the classification performance of the proposed system on differentiating PD patients from the CO subjects.

Positive class (PD)		Negative class (CO)		Specificity (*S*_p_)	Sensitivity (*S*_n_)	Accuracy (*C*_a_)
TP	FN	TN	FP			
13	2	15	1	93.75%	86.67%	90.32%

**Table 5:** Confusion matrix for the classification performance of the proposed system on differentiating HD patients from the CO subjects.

Positive class (HD)		Negative class (CO)		Specificity (*S*_p_)	Sensitivity (*S*_n_)	Accuracy (*C*_a_)
TP	FN	TN	FP			
19	1	15	1	93.75%	95.00%	94.44%

**Table 6:** Confusion matrix for the classification performance of the proposed system on differentiating ND patients from the CO subjects.

Positive class (ND)		Negative class (CO)		Specificity (*S*_p_)	Sensitivity (*S*_n_)	Accuracy (*C*_a_)
TP	FN	TN	FP			
44	4	14	2	87.50%	91.67%	90.63%

**Table 7:** Classification results between the CO group and the two ND groups.

Performance metrics	Results (negative vs. positive)		Mild ND vs. severe ND
	CO vs. mild ND	CO vs. severe ND	
Sensibility	18/21 = 85.71%	27/27 = 100%	23/27 = 85.19%
Specificity	14/16 = 87.50%	16/16 = 100%	17/21 = 80.95%
Accuracy	32/37 = 86.49%	43/43 = 100%	40/48 = 83.33%

**Table 8:** Performance comparison of several state-of-the-art methods for discriminating ND gaits from normal gaits.

	Features	Classifier	Evaluation method	Overall accuracy (%)
ALS vs.CO	Swing-interval turns count; averaged stride interval [1]	LS-SVM	LOO	89.66
	Entropy and coherence extracted from the wavelet approximation of the gait signal [6]	LDA	LOO	86.2
	ANFIS models for left and right stride interval, left and right stance interval, and double support interval (proposed)	Distance rule	LOO	93.10

PD vs.CO	Swing-interval turns count; gait rhythm standard deviation [7]	LS-SVM	LOO	90.32
	Constant RBF networks learned via deterministic learning [12]	Distance rule	LOO	87.1
	ANFIS models for left and right stride interval, left and right stance interval, and double support interval (proposed)	Distance rule	LOO	90.32

HD vs.CO	Entropy and coherence extracted from the wavelet approximation of the gait signal [6]	LDA	LOO	86.10
	Statistical features such as minimum, maximum, average, and standard deviation [30]	SVM	Random subsampling	90.28
	ANFIS models for left and right stride interval, left and right stance interval, and double support interval (proposed)	Distance rule	LOO	94.44

ND vs.CO	Entropy and coherence extracted from the wavelet approximation of the gait signal [6]	LDA	LOO	80.4
	Constant RBF networks learned via deterministic learning [12]	Distance rule	ATAT	93.75
	ANFIS models for left and right stride interval, left and right stance interval, and double support interval (proposed)	Distance rule	LOO	90.63

LS-SVM: least squares support vector machines. LDA: linear discriminant analysis. ATAT: all-training-all-testing. LOO: leave-one-out.

## Data Availability

The data used to support the findings of this study are available at PhysioNet website (http://www.physionet.org/physiobank/database/gaitndd/).

## References

[1] Wu Y., Krishnan S. (2009). Computer-aided analysis of gait rhythm fluctuations in amyotrophic lateral sclerosis. *Medical and Biological Engineering & Computing*.

[2] Hausdorff J. M., Lertratanakul A., Cudkowicz M. E., Peterson A. L., Kaliton D., Goldberger A. L. (2000). Dynamic markers of altered gait rhythm in amyotrophic lateral sclerosis. *Journal of Applied Physiology*.

[3] Nefussy B., Drory V. E. (2010). Moving toward a predictive and personalized clinical approach in amyotrophic lateral sclerosis: novel developments and future directions in diagnosis, genetics, pathogenesis and therapies. *EPMA Journal*.

[4] Goldfarb B., Simon S. (1984). Gait patterns in patients with amyotrophic lateral sclerosis. *Archives of Physical Medicine and Rehabilitation*.

[5] Hausdorff J. M., Cudkowicz M. E., Firtion R., Wei J. Y., Goldberger A. L. (1998). Gait variability and basal ganglia disorders: Stride-to-stride variations of gait cycle timing in parkinson’s disease and Huntington’s disease. *Movement Disorders*.

[6] Baratin E., Sugavaneswaran L., Umapathy K., Ioana C., Krishnan S. (2015). Wavelet-based characterization of gait signal for neurological abnormalities. *Gait Posture*.

[7] Wu Y., Krishnan S. (2010). Statistical analysis of gait rhythm in patients with Parkinson’s disease. *IEEE Transactions on Neural Systems and Rehabilitation Engineering*.

[8] Wu Y., Shi L. (2011). Analysis of altered gait cycle duration in amyotrophic lateral sclerosis based on nonparametric probability density function estimation. *Medical Engineering & Physics*.

[9] Wagenaar R. C., van Emmerik R. E. A. (1996). Dynamics of movement disorders. *Human Movement Science*.

[10] Stergiou N., Decker L. M. (2011). Human movement variability, nonlinear dynamics, and pathology: is there a connection?. *Human Movement Science*.

[11] Liao F., Wang J., He P. (2008). Multi-resolution entropy analysis of gait symmetry in neurological degenerative diseases and amyotrophic lateral sclerosis. *Medical Engineering & Physics*.

[12] Zeng W., Wang C. (2015). Classification of neurodegenerative diseases using gait dynamics via deterministic learning. *Information Sciences*.

[13] Chaoui H., Sicard P. (2012). Adaptive fuzzy logic control of permanent magnet synchronous machines with nonlinear friction. *IEEE Transactions on Industrial Electronics*.

[14] Todorovic N., Klan P. State of the art in nonlinear dynamical system identification using artificial neural networks.

[15] Jang J. S. R. (1993). ANFIS: Adaptive-network-based fuzzy inference system. *IEEE Transactions on Systems, Man, and Cybernetics*.

[16] Jang J. S. R. (1992). Self-learning fuzzy controllers based on temporal backpropagation. *IEEE Transactions on Neural Networks*.

[17] Gorgel P., Sertbas A., Ucan O. N. A fuzzy inference system combined with wavelet transform for breast mass classification.

[18] Übeyli E. D. (2010). Automatic diagnosis of diabetes using adaptive neuro‐fuzzy inference systems. *Expert Systems*.

[19] Gómez C., Hornero R., Abásolo D., Fernández A., Escudero J. (2009). Analysis of MEG background activity in Alzheimer’s disease using nonlinear methods and ANFIS. *Annals of Biomedical Engineering*.

[20] Xia Y., Gao Q., Ye Q. (2015). Classification of gait rhythm signals between patients with neuro-degenerative diseases and normal subjects: Experiments with statistical features and different classification models. *Biomed. Signal. Proces.*.

[21] Hoehn M. M., Yahr M. D. (1998). Parkinsonism: onset, progression and mortality. *Neurology*.

[22] Shoulson I., Fahn S. (1979). Huntington disease: clinical care and evaluation. *Neurology*.

[23] Buragohain M., Mahanta C. (2008). A novel approach for ANFIS modelling based on full factorial design. *Applied Soft Computing*.

[24] Catalão J., Pousinho H., Mendes V. (2011). Hybrid wavelet-PSO-ANFIS approach for short-term electricity prices forecasting. *IEEE Transactions on Power Systems*.

[25] Shoorehdeli M. A., Teshnehlab M., Sedigh A. K., Khanesar M. A. (2009). Identification using ANFIS with intelligent hybrid stable learning algorithm approaches and stability analysis of training methods. *Applied Soft Computing*.

[26] Kennedy J., Eberhart R. Particle swarm optimization.

[27] Xue L., Yin J., Ji Z., Jiang L. A particle swarm optimization for hidden Markov model training.

[28] Yu W., Li X. (2004). Fuzzy identification using fuzzy neural networks with stable learning algorithms. *IEEE Transactions on Fuzzy Systems*.

[29] Vasumathi B., Moorthi S. (2012). Implementation of hybrid ANN–PSO algorithm on FPGA for harmonic estimation. *Engineering Applications of Artificial Intelligence*.

[30] Daliri M. R. (2012). Automatic diagnosis of neuro-degenerative diseases using gait dynamics. *Measurement*.

